# Comparison of Energy Expenditure and Oxygen Consumption of Spontaneous Breathing Trial Conducted With and Without Automatic Tube Compensation

**DOI:** 10.14740/jocmr2250w

**Published:** 2015-07-24

**Authors:** Alessandra Fabiane Lago, Elaine Cristina Goncalves, Elaine Caetano Silva, Mayra Goncalves Menegueti, Edson Antonio Nicolini, Maria Auxiliadora-Martins, Edson Zangiacomi Martinez, Ada Clarice Gastaldi, Anibal Basile-Filho

**Affiliations:** a Division of Intensive Care, Department of Surgery and Anatomy, Ribeirao Preto Medical School, University of Sao Paulo, SP 14049-900 Ribeirao Preto, Brazil; b Department of Social Medicine, Ribeirao Preto Medical School, University of Sao Paulo, SP 14049-900 Ribeirao Preto, Brazil; c Department of Physiotherapy, Ribeirao Preto Medical School, University of Sao Paulo, SP 14049-900 Ribeirao Preto, Brazil

**Keywords:** Indirect calorimetry, Ventilator weaning, Respiratory insufficiency, Artificial respiration, Intensive care units

## Abstract

**Background:**

Weaning from mechanical ventilation is defined as the process of release of ventilatory support and how the evaluation of this phase is conducted in the spontaneous breathing trial (SBT). One of the most used modes of SBT is the continuous positive airway pressure (CPAP), which applies a continuous positive pressure in both inspiration and expiration. However, together with the mechanical ventilation modes, the automatic tube compensation (ATC) can be used, which compensates the resistance imposed by the endotracheal tube. The objective of this study was to compare oxygen consumption (VO_2_) and energy expenditure (EE) during SBT conducted with and without ATC.

**Methods:**

The study was prospective, randomized and crossover. Forty mechanically ventilated patients were admitted to an intensive care unit of a university tertiary hospital. The participants were randomly allocated in group 1, in which SBT was initiated with CPAP and ATC, followed by CPAP without ATC or in group 2, in which SBT was initiated with CPAP without ATC, followed by CPAP with ATC. Indirect calorimetry helped to measure VO_2_ and EE during SBT.

**Results:**

The differences between VO_2_ and EE obtained during SBT with ATC and without ATC were -1.6 mL/kg/min (95% CI: -4.36 - 1.07) and 5.4 kcal/day (95% CI: -21.67 - 10.79), respectively.

**Conclusions:**

We concluded that VO_2_ and EE obtained during SBT with and without ATC were not different.

## Introduction

Weaning from mechanical ventilation refers to the abrupt or gradual withdrawal of ventilator support; it is better designated as “discharge” from mechanical ventilation [1]. Delayed discontinuation of mechanical ventilation might culminate in complications such as ventilator-induced diaphragmatic dysfunction and atrophy, mechanical ventilation-associated pneumonia, and increased morbidity and mortality [2, 3]. On the other hand, early ventilator withdrawal may result in impaired gas exchange and the maintenance of airway permeability, which requires re-intubation [4]. Ventilation discontinuation and withdrawal of the artificial airway are two important aspects of weaning from mechanical ventilation [5]. Evidence-based guidelines recommend conduction of the spontaneous breathing trial (SBT) to evaluate endotracheal tube removal and aptitude of patients for extubation [6].

The SBT can be accomplished with a T piece, in continuous positive airway pressure (CPAP) or with pressure support ventilation (PSV) [7, 8]. Irrespective of the mechanical ventilation mode, the automatic tube compensation (ATC) test can also be employed, to compensate for the resistance imposed by the endotracheal tube. Indeed, this type of resistance can increase the work for inhalation, the rapid shallow breathing index (RSBI), and the pressure-time product (an estimation of oxygen consumption by the muscles of respiration) [9, 10]. ATC involves continuous measurement of flow and pressure as well as adequate selection of tube diameter; it consists of a safe, useful method that can provide the patient with respiratory comfort [11].

Researchers have studied oxygen consumption (VO_2_) and energy expenditure (EE) in mechanically ventilated patients. It was assessed how the variables VO_2_ and EE change upon alterations in mechanical ventilation parameters and modes [12, 13], weaning from mechanical ventilation [14, 15], physiotherapy [16], and nutritional therapy [17-19]. Therefore, VO_2_ and EE constitute gold standard in this field.

## Methods

The research protocol was approved by the Research Ethics Committee of Clinics Hospital, Ribeirao Preto Medical School, University of Sao Paulo, Brazil (protocol 10872/2011).

This investigation consisted of a randomized, controlled, crossover study conducted in an intensive care unit (ICU) of Clinics Hospital of Ribeirao Preto Medical School of the University of Sao Paulo.

Weaning from mechanical ventilation involved gradual reduction in pressure support to 8 cm H_2_O. First, the patient or a next of kin was required to provide an informed consent; then, the inclusion and exclusion criteria were checked. Following these procedures, by means of a draw, a nurse withdrew an opaque, non-translucent, sealed envelope to find out to which group the patient would belong. Neither the main investigator nor the patients had any knowledge of the draw until the beginning of the study. After the draw, demographic and prognostic indices (APACHE II [20] and SAPS 3 [21, 22]) data were collected prior to interventions. The airway resistance was measured at baseline.

### Participants

This study included intubated patients under mechanical ventilation for over 24 h who were apt for SBT. Patients met the following criteria: reversal or control of the event that required invasive ventilation, partial oxygen pressure in arterial blood (PaO_2_) higher than 60 mm Hg, fraction of inspired oxygen (FiO_2_) lower than 0.4, positive-end expiratory pressure (PEEP) lower than 5 cm H_2_O, good clinical signs of tissue perfusion, and presence of spontaneous breathing incursions.

The exclusion criteria included hemodynamic instability, coronary artery diseases, and complex cardiac arrhythmias; water-electrolyte and acid-base imbalance; body temperature above 38 °C or below 35 °C; hemoglobin below 7 g/dL; RSBI under spontaneous breathing higher than 105 cycles/min/L; age less than 18 years; pregnancy; presence of chest tubes; refusal of patient or next of kin to participate in the study; failure during SBT; and agitation.

### Intervention

**Figure 1:**
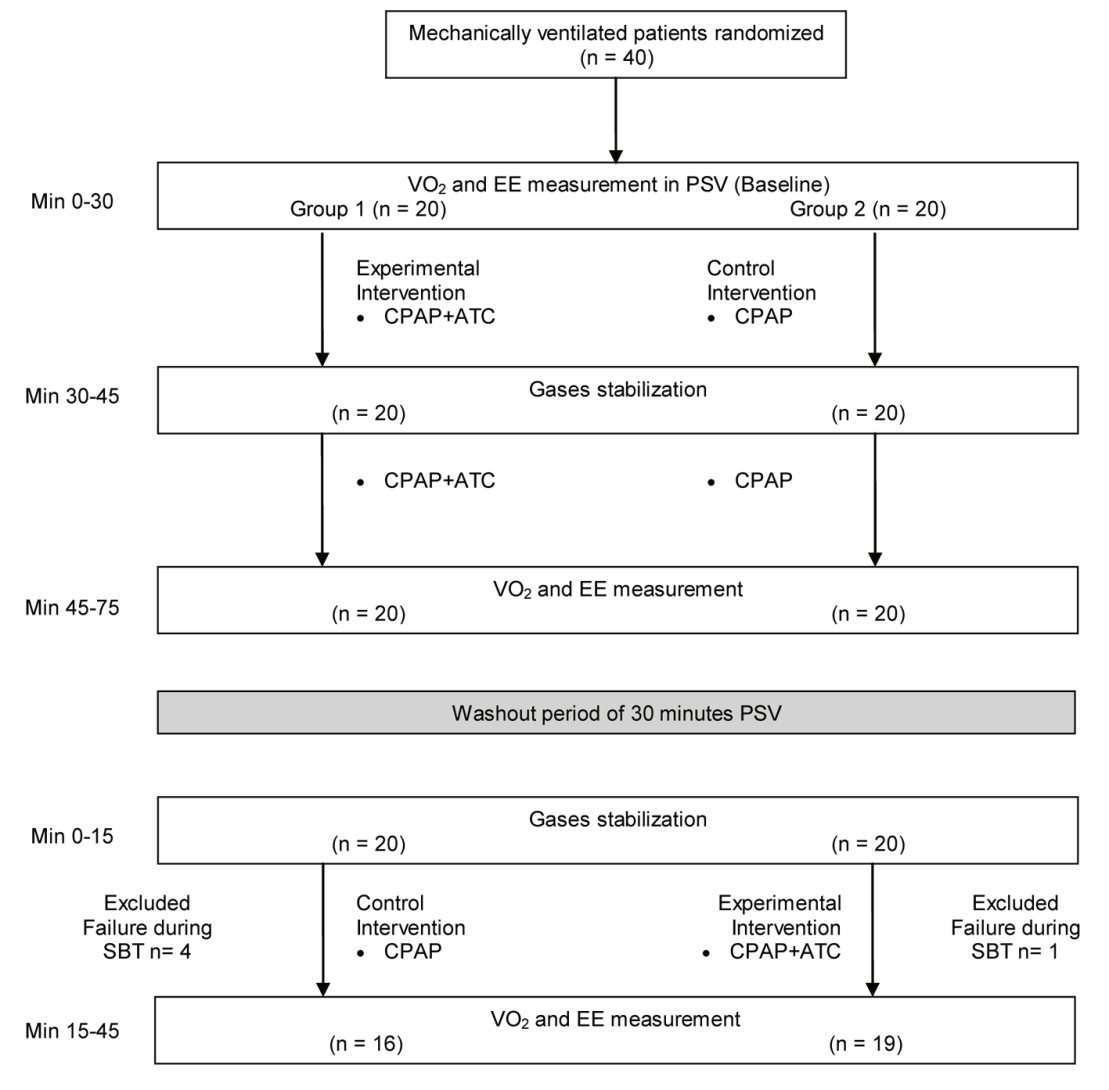
Experimental design used throughout the study.

Figure 1 illustrates the intervention conducted in this study. Group 1 underwent SBT in CPAP associated with ATC (CPAP + ATC) (experimental intervention) followed by SBT in CPAP without ATC (CPAP) (control intervention). Group 2 was submitted to SBT in CPAP without ATC (CPAP), followed by SBT in CPAP associated with ATC (CPAP + ATC). Thirty-minute PSV at 8 cm H_2_O was accomplished between the modes, to eliminate the residual effect of the first procedure during application of the second procedure (washout). In addition, 15 min were allowed between modes, to stabilize the gases.

Both groups had PEEP of 5 cm H_2_O. Oxygen was supplied at the same concentration that the patient had used before SBT. The trigger was set at 3 L/min, with a ramp of 0.2 s and heat and moisture exchanger filter. Before the tests, the patients had been submitted to bronchial hygiene and had been placed in the supine position with the headrest at 30°.

If the patient presented an unfavorable outcome, i.e. respiratory rate higher than 35 bpm, peripheral oxygen saturation lower than 90%, heart rate larger than 140% or 20% variation in basal levels, systolic blood pressure above 200 mm Hg or below 80 mm Hg, sweating, agitation, signs of increased work of breathing as paradoxical respiratory standard, or use of accessory muscles of respiration, the test was interrupted. The patient was submitted to the initial mechanical ventilation parameters, and this event was considered a failure. When these signs did not arise, SBT was deemed successful. If conditions allowed, the patient was extubated.

### Outcome measures

Patients were subjected to VO_2_ and EE measurements with the aid of a calorimeter (DELTATRAC II Metabolic Monitor; Datex-Ohmeda, Helsinki, Finland), for 90 min. First, gas and pressure were calibrated according to the manufacturer’s instructions (calibration gas set to O_2_ = 95% and CO_2_ = 5%). Then, the calorimeter was connected to the mechanical ventilator, and the measurements were performed. The steady-state was defined as 5 consecutive minutes during which oxygen consumption and carbon dioxide production vary less than ±10% as previously described [23].

### Statistical analysis

Data were described through graphic representation of the median, lower and upper quartile, and interquartile range of VO_2_, EE, carbon dioxide production (VCO_2_), and basal respiratory quotient (RQ), with and without ATC. A generalized linear model helped to analyze the dependent variables and the estimated values of the mean of the differences of the two-period change for each effect [24]. The latter were related to the differences in values with respect to treatments, groups, and periods, as follows. Treatment 1 (T1): the SBT was with ATC. Treatment 2 (T2): the SBT was without ATC. Group 1 (G1): treatment started with ATC and ended without ATC. Group 2 (G2): treatment started without ATC and ended with ATC. Period 1 (P1): period before the washout. Period 2 (P2): period after the washout.

The level of significance was set at 5%. Statistical analysis was performed using R version 3.0.1 (2013-05-16) (The R Foundation for Statistical Computing, Vienna, Austria) and SAS version 9.00 (Cary, NC, EUA).

## Results

Recruitment and data collection occurred between June 2012 and September 2013. Fifty-nine patients were selected for the study. Nineteen of these patients were excluded prior to the start of the protocol, six patients had chest tubes, fistula, or leakage, three patients refused to participate in the study, four patients presented RSBI larger than 105 breaths/min/L, and six patients had psychomotor agitation. Five patients were excluded after the beginning of the study, due to failure during SBT; four of these patients belonged to group 1. Therefore, 35 patients participated and completed the study (Fig. 1). The same investigator (AFL) made all the procedures in the study.

**Figure 2:**
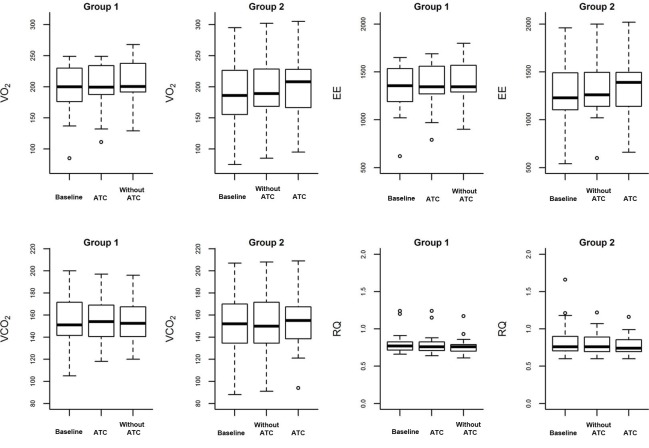
Box plot of VO_2_, EE, VCO_2_, and RQ at baseline, with and without ATC. The diamonds are atypical values (outliers).

Eighteen (51%) of the participants were male. The mean age, the mean APACHE II score, and the mean SAPS 3 score were 61 years (SD 16), 24 (SD 8.4), and 67 (SD 22), respectively. Six patients (15%) died. The mean length of ICU and hospital stay was 14 days (SD 8) and 78 days (SD 71), respectively. Table 1 lists the clinical data; Figure 2 illustrates VO_2_, EE, VCO_2_, and RQ throughout the study. The difference between the averages of the values related during the SBT with and without ATC, group 1 and group 2, and period 1 pre-washout and period 2 after washout are demonstrated in Table 2.

**Table 1:** Study Participants Demographics

Variables	Values
Gender	
Male, n (%)	18 (51)
Female, n (%)	17 (49)
Age (years) (mean ± SD)	61.4 ± 16.1
APACHE II (mean ± SD)	24.6 ± 8.4
SAPS 3 (mean ± SD)	67.7 ± 22
ICU mortality, n (%)	6 (15)
Hospital mortality, n (%)	17 (42.5)
Length of ICU stay (mean ± SD)	14.1 ± 8.2
Length of hospital stay (mean ± SD)	78 ± 70.71
Airway resistance at baseline (cm H_2_O/L/s)	9.02 ± 2.35
ETT size (mm)	7.85 ± 0.49
Reason to initiate MV	
ARF, n (%)	24 (68%)
Septic shock, n (%)	7 (20%)
COPD, n (%)	2 (6%)
Hepatic encephalopathy, n (%)	1 (3%)
Cerebrovascular accident, n (%)	1 (3%)
Cause of ARF	
Pneumonia, n (%)	11 (46%)
Post-operative, n (%)	7 (29%)
Acute pulmonary edema, n (%)	3 (12.5%)
Sepsis, n (%)	2 (8.4%)
Cardiac arrest, n (%)	1 (4.1%)
Diagnosis upon admission	
Septic shock, n (%)	11 (31%)
Respiratory, n (%)	11 (31%)
Post-operative, n (%)	9 (26%)
Cerebrovascular accident, n (%)	1 (3%)
Others, n (%)	3 (9)

SD: standard deviation; APACHE II: Acute Physiology and Chronic Health Evaluation II; SAPS 3: Simplified Acute Physiology Score 3; ICU: intensive care unit; MV: mechanical ventilation; ETT: endotracheal tube or tracheostomy; ARF: acute respiratory failure; COPD: chronic obstructive pulmonary disease.

**Table 2:** Estimated Values for the Differences of T1 and T2, G1 and G2, and P1 and P2

Variables	Estimated value T1-T2	95% confidence interval	P value	Estimated value G1-G2	95% confidence interval	P value	Estimated value P1-P2	95% confidence interval	P value
VO_2_ (mL/kg/min)	-1.6	-4.36 to 1.07	0.23	5.4	-26.44 to 37.43	0.73	-3.5	-6.25 to -0.82	0.01
EE (kcal/day)	-5.4	-21.67 to 10.79	0.500	34.0	-163.79 to 231.93	0.728	-22.8	-39.04 to -6.58	0.007
VCO_2_ (mL/kg/min)	0.3	-2.49 to 3.11	0.82	2.1	-15.30 to 19.64	0.82	1.0	-3.86 to 1.74	0.45
RQ	0.004	-0.01 to 0.02	0.63	-0.007	-0.11 to 0.09	0.88	0.023	0 to 0.04	0.02
Peak pressure (cm H_2_O)	2.00	1.39 to 2.62	0.0001	0.55	-0.93 to 2.03	0.4555	0.35	-0.25 to 0.97	0.2446
Vt (mL)	13.32	-30.65 to 57.30	0.5415	24.20	-89.14 to 137.56	0.6668	17.27	-26.70 to 61.24	0.4296
Rr (bpm)	0.34	-0.87 to 1.56	0.5688	1.26	-2.49 to 5.02	0.4993	-0.76	-1.98 to 0.44	0.2080
RSBI (bpm/L)	1.17	-4.11 to 6.46	0.6531	8.99	-28.32 to 46.31	0.6268	-3.77	-9.06 to 1.51	0.1558
P0.1 (cm H_2_O)	-0.49	-0.84 to -0.14	0.0073	0.14	-1.21 to 1.50	0.8276	-0.39	-0.75 to -0.04	0.0279
HR (beats per min)	-1.84	-3.65 to -0.048	0.0445	-1.38	-9.25 to 12.02	0.7921	-1.41	-3.22 to 12.02	0.1176
MAP (mm Hg)	-0.57	-3.14 to 2.00	0.6526	-7.74	-16.89 to 1.40	0.0942	0.57	-16.89 to 1.40	0.6510
SpO_2_ (%)	-0.11	-0.64 to 0.41	0.6644	-0.63	-1.98 to 0.71	0.3416	0.26	-0.27 to 0.79	0.3235

T1: treatment 1 with ATC; T2: treatment 2 without ATC; G1: group 1; G2: group 2; P1: period 1; P2: period 2; VO_2_: oxygen consumption; EE: energy expenditure; VCO_2_: carbon dioxide production; RQ: respiratory quotient; Vt: tidal volume; Rr: respiratory rate; RSBI: rapid shallow breathing index; P0.1: airway occlusion pressure 100 ms after onset of inspiratory flow; HR: heart rate; MAP: mean arterial pressure; SPO_2_: peripheral oxygen saturation.

## Discussion

Literature comparing VO_2_ and EE by indirect calorimetry in different modes of weaning from mechanical ventilation is scarce. Dos Santos et al [25] compared EE during weaning from mechanical ventilation. These authors randomly allocated 40 patients into two different groups. The first group started weaning in PSV and ended it in T-piece; the reverse order was used for the second group. EE in T-piece was 14.4% higher as compared with PSV. In contrast, in the present study we did not detect any differences between SBT in CPAP with and without ATC in relation to EE.

In another randomized study, Oczenski et al [14] investigated 21 postsurgical cardiac patients without lung alterations under PSV and CPAP with and without ATC. A calorimeter was used to measure the variables and compare the modes of weaning from mechanical ventilation. VO_2_ was 170 (SD 29) vs. 170 (SD 26) vs. 174 (SD 29) mL/min/m^2^ in CPAP with ATC, PSV, and CPAP without ATC, respectively. The authors concluded that patients submitted to heart surgery demanded normal ventilation and did not require ventilator compensation during discontinuation of mechanical ventilation. Here, we compared VO_2_, EE, VCO_2_, and RQ not only between treatments (CPAP with and without ATC), but also between groups and periods, to verify whether these effects influenced comparison between SBT with and without ATC. We only verified differences between VO_2_ and EE values when periods P1 and P2 were compared, the periods during which we applied treatment 1 and treatment 2, respectively. Nevertheless, this difference was not clinically relevant, only 3.5 mL/kg/min and 22.8 kcal/day for VO_2_ and EE, respectively. The treatments, sequences, and periods did not affect SBT parameters obtained with and without ATC.

Based on the studies cited above [14, 25] we decided to use a sample size of 40 patients in the present study.

The data demonstrated that treatment 1 and 2 were equivalent apropos of VO_2_ and EE. Hence, VO_2_ and EE were not different probably since most of the surveyed patients did not present increased airway resistance (9.04 ± 2.35 cm H_2_O/L/s) due to chronic obstructive pulmonary disease (COPD) (only two patients had COPD) or other restrictive conditions.

Cohen et al [7] conducted a randomized, controlled clinical assay on 99 patients to find out whether ATC could minimize the work of breathing during weaning. The authors used CPAP with and without ATC and verified that extubation tended to be more successful during CPAP with ATC as compared with CPAP without ATC, 82% vs. 65%, respectively. Therefore, the use of compensation during the pre-extubation process might be useful.

Later, Cohen et al [26] assessed ATC as a predictor of weaning. The authors compared the use of CPAP combined with ATC at 5 cm H_2_O with the use of PSV at 7 cm H_2_O. The authors observed that RSBI with ATC constituted a good predictor of weaning from mechanical ventilation as compared with the other investigated mode. In the present study, there was no difference in relation to the RSBI when performed the SBT in ATC and without ATC.

In a randomized clinical assay, Figueroa-Casas et al [11] compared patients subjected to weaning from mechanical ventilation under CPAP with and without ATC. These authors concluded that, although ATC was safe, it did not accelerate weaning as compared with CPAP without ATC.

Uyar et al [27] evaluated metabolic variables during PSV and airway pressure release ventilation (APRV). Indirect calorimetry aided analysis of VO_2_, EE, VCO_2_, and RQ. The results from the two methods did not differ from a metabolic standpoint. Finally, the present work had some limitations. First, we calculated sample size based on another literature study. Therefore, the sample size adopted herein might not have been able to evidence differences in metabolic variables, and further investigations might be necessary. Furthermore, it is worth highlighting that other groups of patients, such as patients with chronic obstructive diseases, deserve special investigation. Indeed, these subjects have increased airway resistance at the baseline, and an individual approach in future studies of this particular clinical condition may be interesting if we consider that application of ATC aims to minimize airway work and resistance.

### Conclusion

Finally, in our sample, we concluded that VO_2_ and EE measured by indirect calorimetry during SBT with and without ATC were not clinically different.
